# Post-traumatic knee osteoarthritis; the role of inflammation and hemarthrosis on disease progression

**DOI:** 10.3389/fmed.2022.973870

**Published:** 2022-08-22

**Authors:** Bob J. Evers, Martijn H. J. Van Den Bosch, Arjen B. Blom, Peter M. van der Kraan, Sander Koëter, Rogier M. Thurlings

**Affiliations:** ^1^Department of Experimental Rheumatology, Radboud Institute for Molecular Life Sciences, Radboud University Nijmegen Medical Centre, Nijmegen, Netherlands; ^2^Canisius Wilhelmina Hospital, Nijmegen, Netherlands

**Keywords:** post-traumatic knee osteoarthritis, inflammation, synovitis, hemarthrosis, knee injuries

## Abstract

Knee injuries such as anterior cruciate ligament ruptures and meniscal injury are common and are most frequently sustained by young and active individuals. Knee injuries will lead to post-traumatic osteoarthritis (PTOA) in 25–50% of patients. Mechanical processes where historically believed to cause cartilage breakdown in PTOA patients. But there is increasing evidence suggesting a key role for inflammation in PTOA development. Inflammation in PTOA might be aggravated by hemarthrosis which frequently occurs in injured knees. Whereas mechanical symptoms (joint instability and locking of the knee) can be successfully treated by surgery, there still is an unmet need for anti-inflammatory therapies that prevent PTOA progression. In order to develop anti-inflammatory therapies for PTOA, more knowledge about the exact pathophysiological mechanisms and exact course of post-traumatic inflammation is needed to determine possible targets and timing of future therapies.

## Introduction

Sustaining a knee injury is a known risk factor for the development of osteoarthritis (OA) and major trauma [such as an anterior cruciate ligament (ACL) rupture] leads to post-traumatic osteoarthritis (PTOA) in 25–50% of patients. Whereas, patient with primary OA (of unknown etiology) are generally older, the incidence of knee injury and secondary PTOA is especially high in the younger population due higher incidence of knee injuries in younger patients (1–3). Knee injury leads to reduced mobility and physical health, loss of vitality and a decreased quality of life (1–3). This is most marked in patients that develop PTOA (4). The economic burden of knee injuries and the development of PTOA is substantial (5, 6). The costs attributable to knee injury and PTOA are related to both direct costs, such as medical treatment and rehabilitation and to indirect costs, such as work-related loss (7–9). These losses can be substantial in younger patients. Current treatment of knee injury is aimed at relieving pain and symptoms with non-operative treatment or, when necessary, with surgical repair. Even though surgical repair can increase activity level, the incidence of PTOA is not reduced by surgery (10–14). PTOA accounts for approximately 12% of symptomatic OA patients.

Historically, the pathophysiology of PTOA was believed to result from pathological mechanical stress and abnormal loading of the joint, but an increasing number of studies on both human and animal models has provided strong evidence that inflammation and synovitis play a key role (15–22).

Hemarthrosis is common after knee injury and extensive literature is available on how repetitive joint bleeds in hemophilia patients can lead to cartilage destruction and hemophilic arthropathy (23–26). However, literature on the inflammatory effects of hemarthrosis on the development of PTOA in non-hemophilia is lacking. Several anti-inflammatory and tissue protective therapies have been developed and tested for primary osteoarthritis. These therapies could possibly be of value in the treatment and prevention of PTOA. PTOA is a condition which progresses over a longer period and timely administration of treatment that might decelerate or prevent PTOA progression seems important to decrease the morbidity of the disease and to prevent unnecessary joint replacement surgery (27). More precise knowledge on the natural course of PTOA and the inflammatory effects of hemarthrosis can benefit choosing the right target and timing of these candidate therapies. Therefore, knowledge on inflammation and hemarthrosis in the early phase of PTOA is of importance.

This narrative review focusses on the natural course and clinical aspects of PTOA in the knee joint with a special focus on inflammation and hemarthrosis to summarize the existing literature and to describe gaps in the current knowledge.

## Relevant anatomical structures of the knee involved in post-traumatic osteoarthritis

### Articular cartilage

Articular cartilage is a highly specialized connective tissue overlying the articulating surfaces of joints such as the femoral-tibial and patello-femoral joint providing a smooth and lubricated surface for low-friction articulation and facilitating the transmission of loads with a low frictional coefficient (28). Articular cartilage is, unlike most tissues, devoid of blood vessels, nerves or lymphatics. The dense extracellular matrix (ECM) of articular cartilage, which makes up for ~95% of the tissue volume, is mainly comprised of water collagen and proteoglycans. Chondrocytes, which are sparsely distributed among the ECM, have a largely anaerobic metabolism, have limited cell-to-cell contact and have a low rate of replication (28, 29). Nutrients for the chondrocytes are provided by diffusion from the synovial fluid (28). The structure of the articular cartilage can be divided into four zones with unique properties attributed to each zone. The superficial tangential zone consists of a layer of tightly packed collagen fibers which are orientated parallel to the articular surface and provide resistance to shear forces. The middle zone, in which the collagen fibers are obliquely arranged, marks the transition from resistance to shear to resistance to compressive forces. In the deep zone, the collagen fibers are orientated perpendicular to the articular surface which provides the greatest resistance to compressive forces. The calcified layer forms a firm and secure adhesion to the subchondral bone. Cartilage morphology differs among the articulating surface, with cartilage being thicker in regions where contact pressure is greatest compared to infrequently loaded regions. Morphology and mechanical properties therefore seem to be related to load bearing characteristics (30, 31). This highly specialized and organized structure provides the unique mechanical properties of articular cartilage, but also contribute to the limited intrinsic healing potential once damaged (32).

### Cruciate ligaments

Both the anterior and the posterior cruciate ligaments are situated in the middle of the knee joint and anchor the femur to the tibia. These cruciate ligament have a microstructure that consist of collagen bundles and a matrix made up of proteins, elastin, glycoproteins and glycosaminoglycans (GAGs) (33). Functionally, the ACL prevents anterior translation of the tibia on the femur, and posterior translation is prevented by the PCL (34). The cruciate ligaments are vascularized by branches of the middle genicular artery that enters the joint capsule posteriorly through an aperture in the oblique popliteal ligament (33). Damage to these branches due to rupture of cruciate ligaments lead to intra-articular bleeding also known as hemarthrosis. The proximal part of the ACL is better endowed with blood vessels than the distal part which is poorly vascularized, contributing to the poor healing potential of the ACL (33). The ACL is innervated by the tibial nerve and PCL is innervated by both the tibial and the obturator nerves. Minimal pain fibers are present in the cruciate ligaments and therefore innervation contributes to proprioception of the cruciate ligaments (34).

### Meniscus

Menisci are semilunar-shaped fibrocartilaginous structures sitting on the natural contours of the tibial plateau between the femoral condyle and tibia of the knee and cover one half to two thirds of the articular surface. The meniscus is composed of a dense ECM that consists for the most part of water, collagen and to a lesser extend of GAGs. The cells found within the meniscus are referred to as fibrochondrocytes, as they have a marked resemblance to both fibroblasts and chondrocytes. Fibrochondrocytes synthesize the ECM and meniscal tissue (35). The menisci play an important role in shock absorption and load distribution. Menisci increase the tibiofemoral contact area by 50% in the knee joint and transmit 30–55% of the load in standing position. Menisci also contribute to joint stability, largely as secondary soft tissue restraints to prevent anterior tibial displacement. Compression of the meniscus squeezes liquid out into the joint space, which allows for smoother gliding of joint surfaces, and helps to distribute synovial fluid throughout the joint (36). The outer two-third of the menisci are vascularized by branches of the medial inferior, lateral inferior and middle geniculate arteries. Apart from a perimeniscal capillary plexus that penetrates between 10 and 30% of the meniscus, the inner third of the meniscus is avascular. Additional nourishment is provided by synovial diffusion. The anterior and posterior roots are supplied though endoligamentous vessels (37).

### Synovium

The synovium is a specialized connective tissue that lines and seals the joint cavity and joint fluid from surrounding tissue. The synovium consists of a sublining layer of connective tissue that is rich in collagen, blood vessels lymphatic and nerve fibers and a lining layer containing synoviocytes such as macrophages and fibroblasts. The main function of the synovium are the production of lubricin and hyaluronic acid which contribute to joint lubrication and the maintenance of synovial fluid volume and composition in order to aid in chondrocyte nourishment (19).

## Incidence of knee injuries

The incidence of knee injury ranges from 2.29 to 12 cases per 1.000 inhabitants per year. The age group of patients aged 15 to 24 years accounts for the largest proportion of patients that sustain a knee injury (38–41). Of injuries that contribute to the development of PTOA, cruciate ligament lesions and meniscal lesions are the most common and the extensively studied. The incidence of isolated cruciate ligament injuries was 68.6 per 100,000 person years in the US (42). This is in line with other studies reporting an incidence of 78 per 100,000 inhabitants (43). In female athletes incidence of ACL ruptures is roughly three times greater than in male athletes (44, 45). Estimated incidence of meniscal injuries is reported similar around 60–70 per 100,000 persons, however due to multiple studies demonstrating asymptomatic meniscal tears, true incidence might be underestimated (46–48). Reported concomitant meniscal injury in patients with an ACL tear ranges from one third up to two thirds of patients and consistent findings report higher rates of both meniscal and chondral pathology with increasing time from initial ACL injury to surgical reconstruction due to pathological alterations of kinematics in the ACL-deficient knee (42, 49–54). Higher incidence of meniscal injury has been reported in males (55). The overall quality of the evidence is low and studies on male and female high school and collegiate athletes reported no distinct difference in meniscal injury rate (54, 56, 57).

## Diagnosis of knee injuries

### History-taking after knee injury

A popping sound at the time of injury and direct swelling were shown to have a high positive predictive value of 65% for the presence of positive MRI findings in patients with traumatic knee injuries in primary care (58).

Patients with an acute ACL tear typically present with pain, knee effusion, reduction in knee motion and difficulty to bear weight on the affected knee. Patients with a ACL injury often experience giving way complaints after during activities of daily life or when participating in sports (59, 60).

From history-taking in patients with an meniscal injury, the determinants aged over 40 years, being unable to continue the activity and bearing weight after injury, are associated with a meniscal tear in multivariate logistic regression analysis (61). However, clinical examination was only moderately accurate in diagnosing medial (sensitivity 85–94%, specificity 56–75%) and lateral (sensitivity 66–99%, specificity 66–99%) meniscal injury compared with MRI, according to a systemic review by Brady and Weiss (62).

### Physical examination

Systematic reviews recommend the pivot shift test for ruling in ACL ruptures as it has the highest specificity of all physical tests (94–97.5%) (63). Reports on sensitivity are heterogenous. The anterior drawer test, Lachman test and the lever sign test were reported to have similar sensitivity between 81 and 87.1%. Pooled sensitivity of the pivot shift test was 55% (63, 64).

In diagnosing meniscal injuries clinical examination tests, such as McMurray test, joint line tenderness and the Thessaly test were only moderately accurate for diagnosing medial and lateral meniscal tears and are unable to diagnose or exclude meniscal injuries when performed individually (65, 66).

### Imaging technique

In any acute traumatic knee injury, anteroposterior and lateral knee radiographs are indicated to rule out fractures and associated injuries. On these radiographs, tibial eminence fractures and avulsion fractures involving the lateral aspect of the tibial plateau, also known as Segond fractures, are radiological sings of ACL injuries. When plain radiographs shown no abnormalities, MRI imaging is the most helpful modality in diagnosing ACL injury with reported accuracy of 82–100%. Additional tools in diagnosing ACL tears are knee alignment arthrometers and stress radiographs, but these tools are mostly used for research purposes (67, 68).

In diagnosing meniscal lesions, additional imaging most commonly consists of MRI imaging. Phelan et al. showed that MRI was accurate in diagnosing medial (sensitivity 89% [95% CI 83–94%], specificity 88% [95% CI 82–93%]) and lateral (sensitivity 78% [95% CI 66–87%], specificity 95 % [95% CI 91–97 %]) meniscal tears when using arthroscopy as reference (69).

## Current forms of therapy

Trauma-related ACL and meniscal injuries can be managed non-operative or operatively. Non-operative treatment consists of therapy under supervision from a physical therapist. Arthroscopic surgery is indicated when non-operative treatment fails, and symptoms persists. The decision for surgical treatment is based upon multiple factors including the patient's level of activity, the functional demand placed on the knee, occupation, and the presence of associated injury of other structures in the knee. Injury specific reasons to opt for surgery are joint instability in ACL injuries or locking of the knee in meniscal injuries. Surgery is chosen in most active, younger patients and in high-level athletes. To discuss treatment options, patients should be referred to an orthopedic surgeon (70).

## Course of knee injuries

### Course without surgery

Limited literature is available on the prognosis of non-operatively treated patients with an ACL injury. A decrease of 21% in preoperative vs. postoperative Tegner's score (7.1 to 5.6) has been reported (71) and return to preinjury sporting level is reported to be around 11–19% in conservatively treated patients with an ACL injury (72, 73). The choice on which treatment in ACL ruptures should be based on patient characteristics including the level of physical activity before injury. Conservatively treated patients are therefore less likely to be physically active after injury which might affect treatment goals and introduce bias when comparing the results of operative vs. non-operative treatment.

### Course after surgery

When deciding whether a patient can return to sports, multiple factors should be considered such as timing and type of surgery, patient symptoms, physical examination, isokinetic and functional testing, and information from validated questionnaires on readiness for return to sports (74). A study performed in professional football players showed that 81%-92% of patients returned to some sport after ACL reconstruction, while 65% returned to their pre-injury level (75). A higher return to sports rate of 92% return to some level of sports and 78.6% return to pre-injury level of sports is observed in children and adolescents that received an ACL reconstruction (76, 77).

Graft failure and contralateral tears are reported to occur in 4 to 27% of reconstructed patients with a higher percentage of failure occurring in the first year (74).

Delaying the return to sports for as long as 9 months might be beneficial as it has been shown to reduce the rate of re-injury (78).

Several studies have reported that surgical correction of ACL injury does reduce subsequent meniscal injury and the need for further surgery. These studies also report that surgery does not prevent the development of radiographically evident osteoarthritis (10–14, 79). This suggests that remaining subtle rotational instability, low grade inflammatory processes initiated by knee injury or other currently incompletely understood processes cause PTOA development despite reconstructive surgery.

When PTOA progresses to end-stage osteoarthritis, treatment options are limited to joint replacement surgery (JRS). After JRS patients risk the need for a revision surgery due to fractures, infections, loosening or wear of prothesis components. Since most PTOA develop OA at a younger age than patients with primary OA, the need for JRS in these patients is higher and JRS is indicated at younger age.

## Risk factors for knee injury

Sports activities are a prominent feature in the injured population (38, 40, 42). Approximately 70 to 80% of ACL injuries are the result of non-contact injury associated with landing from a jump, changing direction, or sudden deceleration (74). Several risk factors have been identified to explain non-contact ACL injuries. These risk factors can be divided into different risk categories: extrinsic factors, such as weather condition, playing surface, level of competition, and intrinsic factors such as anatomical, neuromuscular, biomechanical, physiological, psychological, and genetic factors (80). Body weight, fatigue, and neuromuscular deficits such as hamstring strength deficit are modifiable risk factors (80–82). Female gender, age and anatomical features such as decreased femoral intercondylar notch width, increased medial and lateral tibial slopes and ligamentous laxity are non-modifiable factors (67, 80). Failure to correct modifiable factors, such as neuromuscular control and landing biomechanics, increases the risk of injury and recurrence (74) (as summarized in Table 1).

**Table 1:** Risk factors for ACL injury and meniscal injury.

**Modifiable**	**Non-modifiable**
**Risk factors ACL injury**	
**Intrinsic**	**Intrinsic**
>Body Mass Index	Female gender
Neuromuscular deficits	>Age
Landing biomechanics	Increased femoral intercondylar notch width
Fatigue	Increased tibial slope
	Ligamentous laxity
**Extrinsic**	
Weather conditions	
Level of competition	
Type of sport	
Playing surface	
**Risk factors meniscal injury**	
**Intrinsic**	**Intrinsic**
>Body Mass Index	>Age
	Knee malalignment
	Discoid meniscus
**Extrinsic**	Biconcave tibial plateau
Type of sport (soccer, rugby, basketball)	Joint laxity
Occupational activities (squat, kneeling, stair climbing, lifting)	Increased time between ACL rupture and surgery

*ACL, anterior cruciate ligament*.

The risk factors of meniscal injury can be divided into modifiable and non-modifiable factors. Modifiable risk factors are a BMI >25, participation in athletic activities such as soccer, rugby or basketball and occupational prolonged squatting, kneeling, stair climbing and heavy lifting (54, 83). Non-modifiable risk factors for meniscal tears are increased age and anatomical factors such as knee malalignment, discoid meniscus, a biconcave tibial plateau and systemic joint laxity of 1 or more on the Brighton scale (54, 83, 84). Additionally, strong evidence was found for an increased risk on medial meniscal tears when time between ACL injury and reconstruction surgery was >12 months (54, 83, 84) (as summarized in Table 1).

## Pathophysiology of post-traumatic osteoarthritis

**Figure 1:**
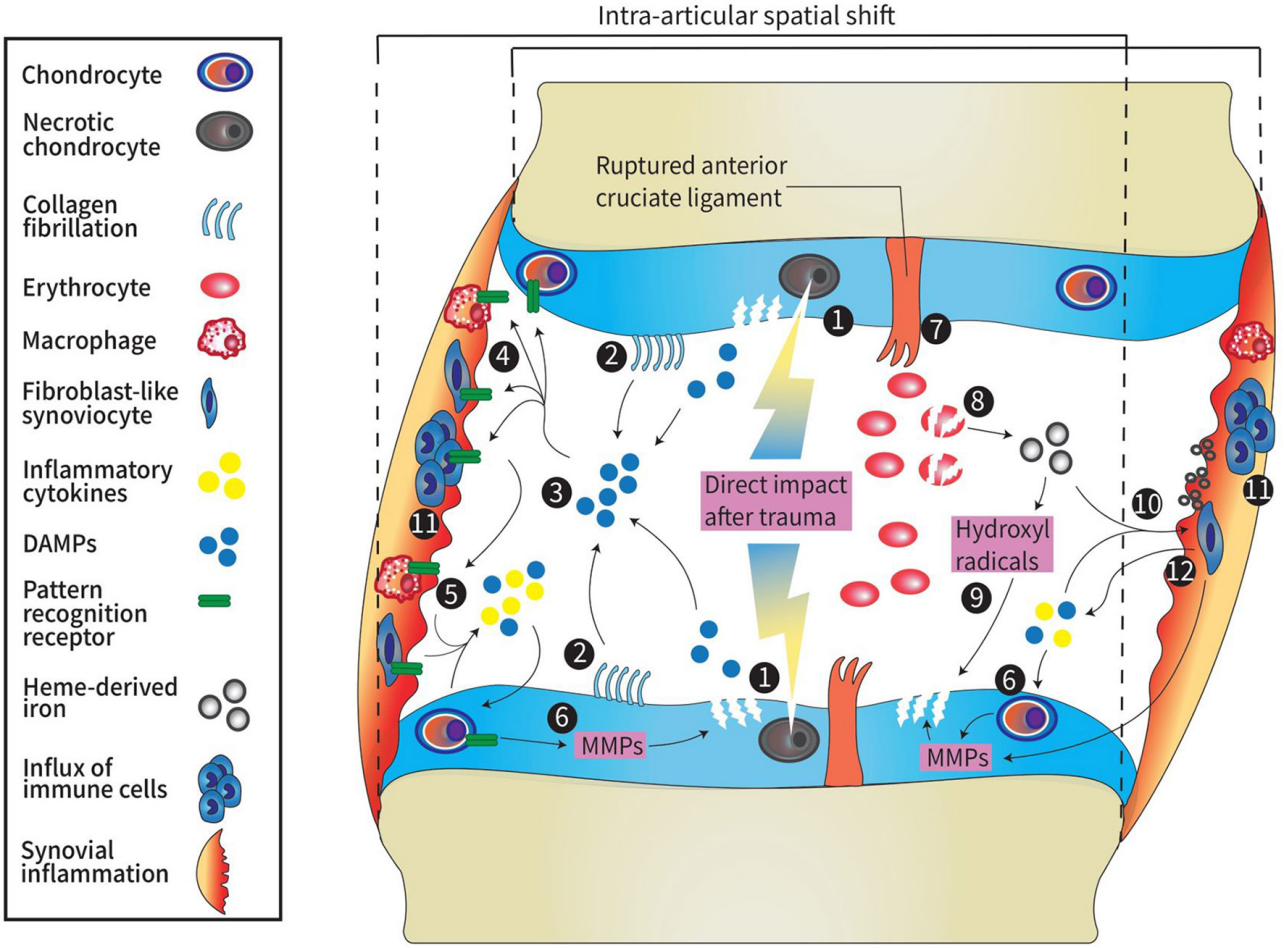
Schematic overview of pathophysiology in PTOA. 1: Direct impact from trauma higher than 10–20 MPa results in necrotic cell death of chondrocytes and damage to the ECM of the cartilage. 2: A spatial shift due to increased joint laxity after ligament rupture causes a change in load bearing contact area. This leads to fibrillation of the collagen network and when fractured, these fibrils act as DAMPs. 3: DAMPs (e.g., glycoproteins proteoglycans, or GAG) are released into the articular cavity as result of acute traumatic cartilage damage. 4: DAMPs bind to pattern recognition receptors (TLRs, NLRs, and RAGE) on the surface of immune cells, chondrocytes, osteoblasts (not shown here), macrophages and fibroblasts. 5: activated cells release inflammatory cytokines such as IL-1, IL-4, IL-6, IL-8, IL-10, IL-17, TNF-α, chemokines, cathepsins, complement cascade factors, and DAMPs [e.g., S-100 proteins, high-mobility group box protein 1 (HMGB1), or uric acid]. 6: Inflammatory factors promote release of MMPs and ADAMTS by synoviocytes and chondrocytes which leads to cartilage degradation. 7: Major peri-articular tissue damage (e.g., anterior cruciate ligament rupture) causes hemarthrosis. Degradation of erythrocytes causes the release of heme-derived iron molecules. 8: Heme-derived iron molecules react with oxygen metabolites generated by monocytes and macrophages forming hydroxyl radicals. 9: hydroxyl radicals induce chondrocyte apoptosis and therefore promote cartilage damage1. 10: Synovial inflammation is induced by DAMPs, pro-inflammatory factors and hemosiderin deposits in the synovium formed by the accumulation of iron molecules. 11: Synovial inflammation is characterized by influx of immune cells. 12: Cells in the inflamed synovium release cytokines, DAMPs and promote release of MMPs leading to cartilage breakdown which again promotes the release of DAMPs into the joint cavity. MPa, millipascal; ECM, extracellular matrix; DAMP, damage-associated molecular pattern; GAG, glycoproteins and glycosaminoglycans; TLRs, Toll-like receptors; NLRs, NOD-like receptors; RAGE, receptor for advanced glycation end-products; IL, interleukin; MMPs, matrix metalloproteinases; ADAMTS 9, a disintegrin and metalloproteinase with thrombospondin motifs.

The pathogenic mechanism of PTOA can be divided into three phases. The first phase is the immediate phase, related to pathological mechanical loading during and after knee injury. The second phase occurs directly after the immediate phase and continues throughout the first weeks after injury. This phase is characterized by an acute inflammatory phase which extracellular matrix fragmentation, cell death and in some cases hemarthrosis occurs. This phase can either resolve spontaneously after a couple of months or persists and progresses into the third phase which is characterized by a chronic inflammatory state with synovitis ultimately contributing to PTOA progression (16). Below, we discuss the pathophysiology of mechanical processes and hemarthrosis that induce inflammation and can lead to chronic synovitis that contributes to PTOA development. A schematic overview of the pathophysiology of PTOA is displayed in Figure 1.

### Pathological mechanical loading caused by knee injury

Uninjured synovial joints are capable of withstanding repetitive loading during normal activities without developing osteoarthritis. However, abnormal joint loading due to injury or joint instability can lead to mechanical demands that exceed the abilities of articular cartilage to repair and maintain itself (85, 86). Immediately after injury, biomechanical changes in cartilage and other joint structures occur. The extent of these changes depends on the severity of the injury (16). Necrotic cell death can occur at the time of injury in areas of impaction >10–20 MPa (86). Collagen rupture due to injury causes cartilage swelling. The collagen damage is irreversible since the rapid loss of tensile behavior cannot be restrained by the collagen network. Loss of cartilage ECM integrity by loss of GAG further predisposes cells to apoptosis (87). Since chondrocytes play an important role in cartilage homeostasis, chondrocyte death will diminish regenerating and repairing capacities of the cartilage and therefore play a central role in PTOA development (16). Blood vessel rupture during and mechanical overload occurring immediately post trauma can cause intra-articular bleeding. Hemarthrosis is thought to contribute to the development of PTOA as it reduces joint lubrication by diluting the synovial fluid, triggers synovitis and can directly cause cartilage damage (16).

Biomechanical alterations after isolated ACL rupture have been extensively investigated by numerous studies and indicate that ACL deficiency alters knee joint kinematics (88–91). In ACL deficient knees, frequently occurring concomitant meniscal injury contributes to altered knee kinematics and further increase joint laxity (92, 93). These biomechanical anomalies lead to altered force distributions on the tibial plateau and a spatial shift of the load bearing area (94).

The knee joint has regional variations in cartilage thickness. A spatial shift from a load bearing contact area to an infrequently loaded area can cause damage to the articulating surface of the cartilage (30, 95). Apart from damage caused by initial trauma, altered chondrocyte metabolism due to changes in loading might also play a role in loss of cartilage integrity (96–98). A presumed pathomechanical model poses that this damage will result in fibrillation of the collagen network and matrix consolidation which in turn increases friction and tangential forces on the articulating surface. The resulting shear stress can lead to fractures of fibrils and upregulate catabolic factors such as matrix metalloproteinase and inflammatory cytokines by chondrocytes (30). Evidence suggesting an association between chondrocyte apoptosis in reaction to these catabolic factors, implies a positive-feedback system when loss of ECM places remaining chondrocytes under greater mechanical and metabolic stress (87, 99).

Bruising of the subchondral bone occurs in approximately 80% of patients who sustain an ACL injury, and its localization is strongly associated with the mechanism of injury (100). Bone bruise are suggested to precede and favor cartilage destruction. Presumed underlying pathophysiological processes include chondral damage due to the initial blow exceeding supraphysiological thresholds. Furthermore, osseous lesions might heal into more rigid callous with decreased compliance and may therefore cause overlying cartilage to absorb more of the load force leading to progressive degeneration of the articulate surface (101). However, no clear correlation between the presence of bone bruises and functional outcome has been indicated in previous studies. Therefore, long term follow-up studies are needed to better determine the clinical significance of bone bruises in the development of PTOA (102).

### The role of hemarthrosis in PTOA development

Reported incidence of acute traumatic hemarthrosis is 4.7 per 10.000 inhabitants (103). In patients that present themselves at the emergency department with rapid swelling of the knee after injury hemarthrosis was confirmed by aspiration of blood in 53% of patients. The most common structural injury in patients with hemarthrosis was ACL rupture (52%) followed by meniscal tear (41%), lateral patellar dislocation (17%) (104). Hemarthrosis is studied to a large extent in patients with hemophilia and can lead to deterioration of the state of the affected joint (105). Inflammatory markers are elevated in the synovial fluid of patients with traumatic-hemarthrosis shortly after knee injury (21). Therefore, hemarthrosis is believed to further contribute to the development of PTOA. The assumed pathogenesis in which joint hemarthrosis promotes inflammation is related to the breakdown of erythrocytes that results in the release of heme- an iron-containing complex. Interaction between this heme-derived iron with oxygen metabolites generated by monocytes and macrophages, namely hydrogen peroxide, results in forming the deleterious hydroxyl radical. The deleterious hydroxyl radical plays an important role in pathogenic chondrocyte apoptosis (106, 107). Heme-derived iron from erythrocyte breakdown accumulates and forms synovial hemosiderin deposits when it is taken up by synoviocytes and macrophages. These heamosiderin deposits are considered to promote synovial inflammation and induce synovial tissue to produce pro-inflammatatory cytokines (24, 108, 109). Furthermore, increased MMP activity and irreversible inhibition of proteoglycan synthesis, even under low concentrations of blood exposure to articular cartilage, leads to unfavorable changes in the extracellular cartilage matrix (110, 111). Other studies have shown release of the pro-inflammatory cytokines IL-1β, TNF-α, and IL-6 after exposing healthy human cartilage to 50% v/v blood for up to 10 days (112, 113). These cytokines are known to play an important role in cartilage matrix degradation (114). Taken together, these processes lead to an imbalance of the hemostasis of the joint resulting in inflammatory changes of which decreased range of motion secondary to pain, synovial hypertrophy and reactive blood vessels are clinical evidence of Potpally et al. (107).

The optimal treatment of traumatic hemarthrosis is under debate. Generally, hemarthrosis is not aspirated unless there is significant swelling or unmanageable pain or a suspicion of infection (107, 115). Conservative non-operative management, including cooling, compression and immobilization is generally the treatment of choice (116). For blood evacuation physicians rely on natural mechanisms to self-resolve the hemarthrosis. Arguments against the routine aspiration of hemarthrosis are the invasive character of the procedure, the chance of complications such as the development of a septic joint infection by introducing bacteria in the joint space, and the need for anesthesia in most of the pediatric population (117, 118). However, the incidence of iatrogenic infections can be lowered when sterile technique is performed by experienced physicians (107). Arguments in favor of aspiration of hemarthrosis are pain relief and greater sensitivity of physical examination maneuvers due to greater range of motion shown by a study evaluating the efficacy of aspiration in the emergency department 2 week after injury (119). Furthermore, animal studies have shown that complete resorption of blood from the joint space takes at least four days and therefore timely aspiration of blood has been suggested as means of minimizing the potential cartilage damage (110, 120).

The induced state of chronic inflammation with cartilage breakdown as a result, might contribute to the development of post-traumatic osteoarthritis. However, longitudinal data on course of inflammation in synovial fluid of joints with traumatic hemarthrosis is missing.

### Acute inflammation of the knee joint after injury

Damaged joint tissues result in the release of damage associated molecular patterns (DAMPs) to the joint cavity. DAMPs are endogenous stimuli that are released either from ECM (e.g., glycoproteins proteoglycans, or GAG) or dying cells [e.g., S-100 proteins, high-mobility group box protein 1 (HMGB1), or uric acid]. DAMPs bind to pattern recognition receptors including injury Toll-like receptors (TLRs) NOD-like receptors (NLRs) and the receptor for advanced glycation end-products (RAGE) which are found on the surface of immune cells, chondrocytes, osteoblasts, macrophages and fibroblasts. This binding activates downstream signaling cascades leading to the release of inflammatory cytokines by synovial cells such as IL-1, IL-4, IL-6, IL-8, IL-10, IL-17, and TNF-α that are found in increased levels in traumatized joints (27, 121–123). Other inflammatory factors that are produced by synovial cells are chemokines, cathepsins, and complement cascade factors. These factors promote deleterious effects on joint cartilage and activate chondrocytes and synoviocytes to produce degradative matrix metalloproteinase (MMPs) and a disintegrin and metalloproteinase with thrombospondin motifs (ADAMTS) 4 and 5. Since these degrative enzymes take part in cartilage breakdown which further stimulates synovial inflammation a vicious circle can arise (124). In addition, cytokine and cell adhesion molecules induce an influx of leucocytes in the synovial tissue and joint cavity. No detailed studies on synovial cell composition early after trauma have been performed but several studies have shown macrophages, which can cluster and form multinucleated giant cells, and T-cell lymphocytes to be the most predominant immune cell in OA synovium (19, 125, 126). The exact role of T-cells in OA progression is not yet fully understood. Once the infiltrated immune cells, such as macrophages, release pro-inflammatory cytokines in order to clean up and remove debris before remodeling can occur. However, since repair of cartilage by chondrocytes is too slow to overcome the induced degradation and damage from injury, inflammation modulating cells may never get an adequate signal to stop this cleanup process which contributes to the before mentioned vicious circle (17, 127).

### Chronic inflammation and synovitis after knee injury

Stiffness, joint pain, and swelling are clinical sings of synovitis that is commonly seen in primary OA and is not limited to end stage OA but can be present in early stages OA as well (19). In the early 1980's abundant inflammation was demonstrated during histopathological analysis of OA synovium in the majority of OA patients (128). Synovitis prevalence of 67% in synovium was shown in end stage OA surgeries compared to 11% in post-mortem control patients with no OA history or pain (129). The incidence of synovitis varies with different imaging modalities and increases with radiologic OA severity. For example, when using ultrasound detection of synovitis inflammation or effusion prevalence varies between 33 to 52%. However, in 125 patients with mainly moderate to severe OA synovitis was seen in 95% on CE-MRI (19). Both animal and human studies have shown that knee injury such as ACL rupture or meniscal lesions can cause synovitis (15, 130, 131). This is supported by observations of prolonged elevation of inflammatory cytokines up to years after knee injury (27).

Macroscopic features of synovitis consist of the presence of vascularity, villi, fibrin deposits and hyperplasia. Microscopic histological features consist of synovial hyperplasia, extent of inflammatory infiltration and stromal cell activation (130, 132). Synovitis can been seen in suprapatellar, infrapatellar, lateral and medial parapatellar and subpopliteal locations, as well as adjacent to posterior cruciate ligaments and severity can vary among these anatomical locations in the knee joint, resulting in a patchy distribution (133).

Abundant infiltration of leukocytes is observed in synovial tissue of both traumatic and non-traumatic OA patients, consisting of proliferation of fibroblast-like synoviocytes (FLS) and macrophage infiltration of the synovial lining. T-cells and to a lesser extent, B cells, mast cells, plasma cells and endothelial cells as components of blood vessels can be found in the synovial sub-lining (19, 126, 130). Synovial cells will phagocytize products of cartilage breakdown that are released into the synovial fluid. When activated in turn, FLS will secrete cytokines, growth factors, MMPs and tissue inhibitors of metalloproteinases (TIMPs) which will contribute to macrophage activation and stimulate catabolic pathways in chondrocytes. The inflamed synovium also contributes to the formation of osteophytes by production of TGF-beta and bone morphogenetic proteins (BMPs) by synoviocytes and macrophages (19, 130, 134, 135).

Studies examining the independent effect of synovitis on non-traumatic OA in the MOST cohort showed an association between synovitis and incident OA after adjusting for confounding pathologies when total synovitis score was 3 or higher (OR 1.6, 95% CI 1.2–2.1) (136). In the same cohort, increased risk of OA development was found in knees without radiographic or non-CE-MRI defined cartilage damage when synovitis was present (OR 2.7, 95% CI 1.4–5.1) (137). The association of synovitis prior to incident OA is further supported by other studies (138–140). In knees with apparent OA, presence of synovitis is associated with a higher risk of radiographic progression of OA (19). Synovial inflammation is associated with clinical symptoms (141, 142). The responsiveness of peripheral nociceptive neurons is increased by immune cells such as synovial CD4+T cells and cytokines such as TNF-α and IL-6. This causes an increased pain experience (19). As mentioned before, synovitis is distributed in a patchy fashion in the knee joint and distinct patterns of synovitis might a cause different pain experience (143). These findings on structural progression and clinical symptoms add to the increasing body of evidence that suggests the importance of low-grade inflammation in OA progression in non-traumatic patients. However, how inflammatory biomarkers and MRI-defined synovitis are associated with patient reported outcomes and structural evidence of OA development in post-traumatic patients is less clear (144, 145). Previous studies showed MRI-defined synovitis and inflammatory biomarkers did not predict structural OA or patient reported outcomes 5 years post ACL injury (144, 145). However, these studies did not investigate if excessive inflammation, hemarthrosis early after trauma or recurrent inflammatory flares were determinants. Additional studies with more detailed analyses early after trauma, with close and longer follow-up are needed to better define the role of inflammation on progression of OA after knee injury.

## Other contributing factors in PTOA development

Patients with malalignment of the knee have an increased risk of developing knee osteoarthritis (146). A possible explanation is that joint malalignment contributes to synovial inflammation. Synovial inflammation decreases in patients that receive extra-articular correction of varus malalignment of the knee join (147–149). This shows that inflammation is induced by pathological loading of the knee cartilage and contributes to a negative feedback of inflammation and cartilage destruction.

Obesity and overweight are well-known risk factors for the development of OA. Numerous studies have shown an increased incidence of radiographic OA in obese patients after ACL reconstruction, indicating that obesity contributes to the development of PTOA (150). Interestingly, an increased incidence of hand OA is found in obese patients and the underlying mechanism is therefore not exclusively explained by mechanical effects (151). Obesity, particularly abdominal obesity, is associated with the metabolic syndrome (152). The systemic effects of metabolic and inflammatory pathways in patients with the metabolic syndrome, such as adipose tissue inflammation, high fasting blood glucose, dysregulated lipid homeostasis and vascular inflammation are believed to cause a low-grade inflammatory state with elevated levels of adipokines that are involved in damage to peripheral tissues, including joint tissue (153, 154). Furthermore, increased prevalence and severity of synovial inflammation on MRI-imaging was seen in obese patients with knee OA, compared to non-obese patients (155). The metabolic syndrome might be an independent risk factor as OA was found to be more prevalent in obese women with cardiometabolic defects compared to obese women without cardiometabolic defects (156).

## Future anti-inflammatory therapies

Prolonged inflammation after knee injury can induce a vicious circle of inflammation and damage to joint tissue. Therefore, anti-inflammatory therapies have been proposed as a potential therapy to break this vicious circle. The most studied anti-inflammatory therapies in OA are inhibitors of IL-1, IL-6, and TNF-α. To date only a limited number of clinical trials have tested anti-inflammatory agents in human patients to impede PTOA of the knee (157). The only published trials are a proof-of-concept RCT using anakinra in 11 patients with an ACL rupture and a RCT using corticosteroid or placebo injection in 49 patients with an ACL injury. Both RCT's reported on short-term patient reported outcomes and biomarkers with anakinra showing improvement in patient reported outcomes and corticosteroid injections showing improvements in some chondrodegenartive biomarkers (158, 159). Multiple promising targets to counteract post-traumatic inflammation exist, leaving room for future clinical trials (157).

Cell-based therapy has been studied as another therapy that may prevent the development of PTOA. Due to their simple acquisition, rapid proliferation, and hypo-immunogenic nature mesenchymal stromal cells (MSC) and adipose stromal cells (ASC) are the most extensively studied cell-based therapies (160). Experimental animal models of post-traumatic OA have shown that intra-articular injection of adipose-derived stem cells can decrease joint inflammation and ameliorate OA if injected under inflammatory conditions (161). When exposed to pro-inflammatory cytokines MSC release anti-inflammatory and immunomodulating proteins. Macrophage polarization to pro-resolving M2-macrophages by MSC and ASC are observed *in vitro*. Even though the underlying mechanisms are not completely understood, cell based therapy using MSC and ASC are promising in OA treatment and prevention (162). Patients with a history of ACL rupture or meniscal lesions are potential suitable patients for monitoring of PTOA onset since a clear starting point of disease onset is usually present. This makes PTOA patients uniquely suitable for early administration of cell-based therapy's when inflammatory disease onset of PTOA becomes apparent in order to prevent progression. However, more knowledge on the exact course of inflammation and possible toxicity of hemarthrosis on cell-based therapy is needed to determine optimal timing of administration of these therapies.

## Conclusion

PTOA is common condition that causes a decrease in quality of life and an immense economic burden on global health care. Current treatment modalities fail in preventing PTOA after significant knee injury. Knowledge on both mechanical and inflammatory processes that play a role in the development of PTOA is growing, but the pathophysiology that causes PTOA is not yet fully understood. Hemarthrosis presumably contributes to the induction of an inflammatory state in the knee joint after knee injury and therefore plays an important role in the development of PTOA. More knowledge on the course of inflammation shortly after injury, the inflammatory effects of hemarthrosis and the effect of hemarthrosis on promising therapies, such as cell-based therapy, is of the utmost importance to progress to more effective therapies for (young) patients at risk of developing PTOA.

## Author contributions

BE wrote the first draft of the manuscript. All authors contributed to manuscript revision, read, and approved the submitted version.

## Funding

This review represents independent research part-funded by Health Holland and the Dutch Arthritis Association.

## Conflict of interest

The authors declare that the research was conducted in the absence of any commercial or financial relationships that could be construed as a potential conflict of interest.

## Publisher's note

All claims expressed in this article are solely those of the authors and do not necessarily represent those of their affiliated organizations, or those of the publisher, the editors and the reviewers. Any product that may be evaluated in this article, or claim that may be made by its manufacturer, is not guaranteed or endorsed by the publisher.
